# Ectopic pregnancy at the Gambian Tertiary hospital

**DOI:** 10.4314/ahs.v21i1.38

**Published:** 2021-03

**Authors:** Matthew Anyanwu, Grace Titilope

**Affiliations:** 1 Edward Francis Small Teaching Hospital (EFSTH), Banjul The Gambia; 2 School of Medical and Allied Health Sciences, university of The Gambia; 3 College of Medicine American International University West Africa

**Keywords:** Ectopic, pregnancy, incidence, risk factors

## Abstract

**Background/Aims:**

Ectopic pregnancy is a gynaecological emergency with significant burden of maternal mortality and morbidity in the tropics. The incidence reported in the literature range from 1:60 to 1:250 pregnancies. The aim was to determine incidence and risk factors of ectopic pregnancy in the Gambia.

**Methodology:**

A longitudinal study of ectopic pregnancy at Gambian tertiary hospital from January 2016 to April 2018. Data was collected from patients' folders, entered into SPSS version 20 and analysed with descriptive statistics. The test of variation and significance was by ANOVA and Chi-square respectively with error margin set at 0.05 and confidence interval of 95%.

**Results:**

A total number of 2562 pregnancies were recorded, 43 were ectopic pregnancies. The estimated incidence was 0.2%. Majority of the patients were between 26 – 35 years (56%), primiparous (32%), heterogeneous marriage (82%) and housewives (86%). Occupation was not associated with ruptured or unruptured ectopic pregnancy (p-0.421). Low parity was associated with more ectopic pregnancy than high parity (p-0.001). The commonest clinical feature was abdominal pain (65.1%), whilst the most prominent risk factors were pelvic inflammatory disease (27.9%) and previous abortion (23.3%). Ectopic pregnancy was seasonal.

**Conclusion:**

The incidence rate of 0.2% was in the range reported in the literature. Low parity, previous abortion and pelvic inflammatory disease were the risk factors.

## Introduction

Ectopic pregnancy (EP) is the abnormal implantation of a conceptus outside the normal endometrial lining of the uterus^1^. It could also be defined as a pregnancy in which implantation occurs in a site other than the uterine endometrial lining. It is a very important gynaecological emergency and a major contributing factor to the burden of maternal mortality and morbidity in the early half of pregnancy^2^.

The incidence of ectopic pregnancy is increasing worldwide and varied from 1 in 60 to 1 in 250 pregnancies^1^. This wide variation has been attributed to degree of genital tract pathologies and contraceptive practice of the population^3^. In sub-Saharan Africa the incidence of ectopic pregnancy is much more difficult to determine especially where maternal deaths that occur due to ectopic pregnancy are not frequently reported or documented^4^.

It is a known fact that ectopic pregnancy (EP) contributes immensely to maternal mortality especially in the tropics. Therefore, efforts to reduce cases of ectopic pregnancy; ensure early diagnosis and appropriate management is crucial in reducing maternal mortality due to ectopic pregnancy. The sustainable development goal 3 states that global maternal mortality ratio should be less than 70 per 100000 births, with no country having a maternal mortality rate of more than twice the global average^5^. Therefore, reducing deaths from EP is one essential element towards realization of this goal.

The occurrence of ectopic pregnancy has in part been linked to the presence of risk factors although some may occur without any risk factor documented. Overall, the risk factors implicated were previous ectopic pregnancy, pelvic inflammatory disease, multiple sexual partners, previous tubal surgeries, intrauterine contraceptive device, smoking, in vitro fertilization, infertility, vaginal douching, migration of the ovum to the contralateral tube, sexually transmitted disease, maternal age, kinking or scarring of the fallopian tube, prior induced abortion, delayed ovulation, and abdominal surgery^6^–^9^. Some other scholars working elsewhere have included consanguinity as a risk factor in women less than 20 years of age^10^,^11^. Congenital malformation of the fallopian tubes due to exposure to diethylstilbestrol is also a risk factor^12^.

The difficulty in reaching a diagnosis has been a contributing factor in the adverse outcome of ectopic pregnancy. The late arrival to hospital and delay in reaching a diagnosis due to multiple factors in the tropics continue to dominate the terrain. In reaching a diagnosis; clinical history, serum human chorionic gonadotrophin (hCG) and a transvaginal scan in combination as a triad is essential. Effective application of the triad certainly may reduce the dilemma. However, laparoscopy can be used for both diagnosis and treatment but is no longer the gold standard of reaching a diagnosis^13^.

Late diagnosis is a risk factor of ruptured ectopic which may be associated with torrential loss of blood. Therefore, haemorrhage is the major reason why it is a leading cause of mortality in women with or without vaginal bleeding which may be subtle or massive. At times vaginal bleeding may not be seen as patient may present in shock and death may result within a very short span of time.

In the Gambia, ectopic pregnancy is still a cause of death as haemorrhage remains the leading cause of maternal mortality^14^. Therefore, the knowledge on the risk factors and incidence of ectopic pregnancy in the Gambia may inform practice and develop a baseline for estimation of trend and its role in the rate of maternal mortality.

## The methodology

A retrospective longitudinal study of ectopic pregnancy from January 2016 to April 2018 was conducted at Edward Francis Small Teaching Hospital, the only teaching hospital and tertiary referral hospital in the country. It is located in Banjul.

**Study population:** All patients with early pregnancy issues seen at the gynaecology clinic or ward were identified but only those with ectopic pregnancy as a diagnosis was considered for analysis during the period under review. Women beyond the child bearing age and those without pregnancy related issues were excluded from the study.

**Procedure:** A longitudinal descriptive study of ectopic pregnancy at the gynaecology inpatient and outpatient department of Edward Francis Small Teaching Hospital (EFSTH). Data was collected from patients' folders after going through the ledger books recording total number of patients in the period under review and their hospital numbers through which the folders were traced. The denominator of the incidence estimation was the number of women in the reproductive age that had confirmed pregnancy during the period under review.

A data collection tool was developed with variables designed to assess the objectives of the study. The variables include; socio-demographic characteristics of the study population, suggested risk factors from the literature review, use of contraceptive and clinical features. Consistent check was used to ensure accurate data entering into SPSS version 20 software. The results were expressed in descriptive statistics by simple percentage. The test of variation and significance was by ANOVA and Chi-square respectively with error margin set at 0.05 and confidence interval of 95%.

## Results

During the period under view (January 2015 to April 2018) a total number of 2562 pregnancy was recorded out of which 43 were ectopic pregnancies. The estimated incidence rate was 0.2%.

The analysis revealed that most of the study population was aged between 26–35 (56%) years which can be categorized as young adults in the study area. The result of the test of significant revealed (F = 8.940, p=0.000<0.05) which inferred a significant difference in the age of the study population. Majority were in heterogeneous marriage (82%). The distribution of ethnicity showed Jola (27.9%) and Mandinka (25.6%) being the most affected tribes.

Regarding occupation, the majority (86.0%) were housewives.

Pelvic inflammatory disease (27.8%) and previous induced abortion (23.3%) were the most common risk factors. In 17 patients (39.5%) no risk factor was stated in the folder.

The Chi-Square analysis (ϰ^2^ = 0.48, p=0.421>0.05) indicates no significant association between occupation and ruptured or unruptured ectopic pregnancy. Ruptured EP accounted for 34 (79.01%) and unruptured EP accounted for 9 (20.09%).

ANOVA analysis (F=172.4, p-value <0.001< 0.05). There was significant association of low parity and ectopic pregnancy. Similarly, there was significant difference or variation in the age and frequency of ectopic pregnancy. The age group of 26–35 was significantly associated with ectopic pregnancy (f = 23.203, p=0.000<0.05).

Distribution of clinical presentation of the patients showed that majority (65%) presented with abdominal pain as the primary complaints. This may or may not be associated with bleeding (16.3%), amenorrhea (14%) or collapse (5%).

This showed that most of the patients were referred (90.7%).

The trend of ectopic pregnancy for a 2-year period from 2016, 2017 and part of 2018; showed sharp increase consistently between February and March. A second increase was from July to October before a sharp fall in December for all the years. The double peak trend observed was in cold and rainy seasons of the Gambia.

## Discussion

The incidence of ectopic pregnancy in this study was 0.2% of all pregnancy during the period under review. This incidence was lower than figures reported in North-East Nigeria (0.6%), India (0.6%), Ghana (2.5%), Saudi Arabia (0.58%) and Southern Nigeria (3.3%)^11^,^5^,^3^,^15^,^16^. The incidence of ectopic pregnancy showed a world-wide variation as the Western worldthe United Kingdom (0.01%) and the United States of America (0.02%)^17^,^18^ had very low incidence of ectopic pregnancy which was comparable with 0.02% incidence recorded in the North-West (Sokoto) part of Nigeria^19^. This discovery made it much more difficult to conclusively offer the reason for this discrepancy which is also beyond the scope of this study suggesting a research interest for the future.

However, wide variation of incidence has been attributed to degree of genital tract pathologies and contraceptive practice of the population^3^.

The intra-country variation of incidence was obvious in Nigeria context where three regions showed different incidence^11^,^16^,^19^. All were hospital based studies but with potential differences in the degree of genital tract pathology, contraceptive practice, culture, religion and socio-economic strata. The impact of these factors may be explored further suggesting a research interest of the future. Also we observed in the studies that different denominators were used which will further make comparison extremely difficult^11^.

In our study we recorded 0.2% incidence which was in the middle of world-wide variation of incidence of ectopic pregnancy (EP). The Gambia has wide antenatal care coverage where 94% of the pregnant mother attends antenatal care at least once and many (75%) achieve 4 visits, however, 18%^20^ of women delivered in health facilities country wide. The low hospital delivery may not affect incidence of ectopic pregnancy as 95% occur in the fallopian tubes which may not accommodate pregnancy to point of delivery. What was pertinent and not exclusive to the Gambia is cultural and religious reasons that discourage autopsy as many women who presented with abdominal pain in the health facilities may die with EP undiagnosed.

The majority of EP recorded was ruptured (79%) which suggests late diagnosis.

**Fig 2:**
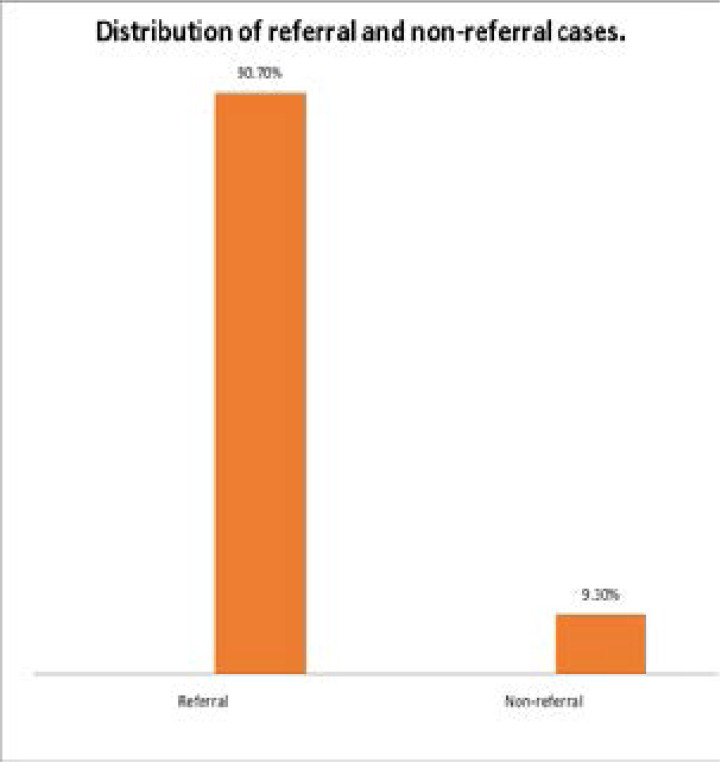
shows distribution of referred and non-referred cases

Poor health seeking behaviour associated with ignorance and poverty may have contributed to late presentation but institutional factors (inadequate manpower, limited availability of consumables, equipment and appropriate test kits) may have also contributed to late diagnosis in this series as these women may have visited a health centre before being referred (91%) (fig 2) to our tertiary hospital with ruptured EP (79%).

Occupation of the patients had no significant association to ruptured or unruptured EP (p-0.421). Most of the women were housewives 86% and 14% of the women were government workers.

Regarding ruptured EP (79%) our finding was similar to Obed et al^21^ where 5.43% cases were unruptured EP. Other studies conducted elsewhere recorded more than 70.1% cases of ruptured EP^22^,^23^,^24^. Ikaki et al^25^ recorded 92.8% cases of ruptured EP and Ekinne et al^26^ recorded 96.2%.

About 56% of the patients were between 26–35 years and were mainly of low parity and about 82% were married. This was comparable with the findings reported in Volta- Ghana, Nigeria, and Senegal^3^, ^11^, ^19^, ^27^. The peak reproductive age falls within these age range of 21–35 years especially in the Gambia. A woman who has low parity compared to women who has high parity is more likely than not to have ectopic pregnancy p-0.001. This was consistent with the recent evidence that ectopic pregnancy is increasing in young women of low parity^28^,^29^. Low parity may be more strongly associated with EP because of the use of certain contraceptives which was not part of this study. However, Ahamed et al^30^ found associations between the occurrence of ectopic pregnancy and low parity with the use of intrauterine contraceptives.

**Figure 1:**
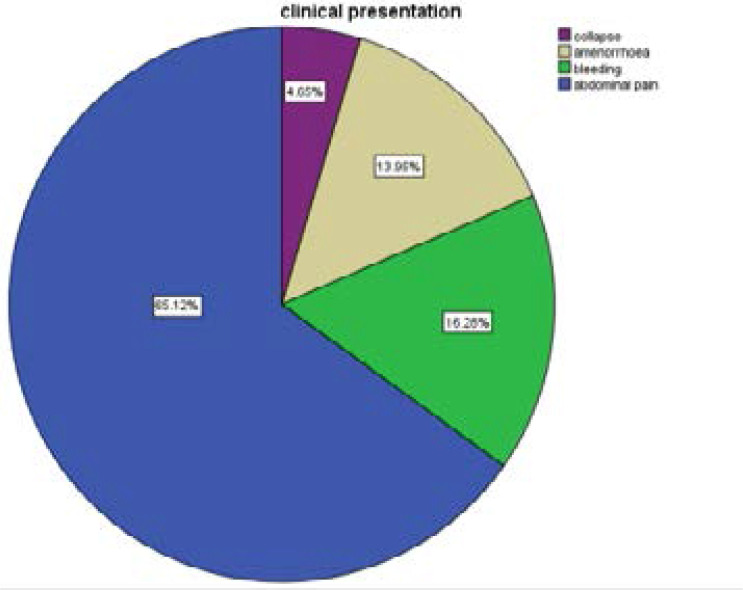
Clinical presentation of the patients as stated in the folders

More than half of the patients (65.1%) presented with history of abdominal pain in addition to bleeding per vaginam (16.2%) and history of amenorrhea (13.9%) (fig 1). This was similar report from other countries like Nigeria, and Ghana^11^, ^3^. No patient presented asymptomatically or with features of shock as seen in Nigeria reports11. However, scholars working in Ireland Chudhary et al^31^ reported cases of EP that presented with shock. It can then be said that a patient that presents with abdominal pain, bleeding and amenorrhea should be triage immediately and considered to have ectopic pregnancy (EP) until proven otherwise.

The main risk factors the women had was pelvic inflammatory disease (27.9%) which was not comparable with Smitre et al^32^ where previous tubal surgery accounted for 40% of women who presented. Induced abortion 95.9% was found to be highest risk factor in South-South Nigeria^16^. Other studies conducted in the sub-region recorded high rates of pelvic inflammatory disease as the common risk factor^23^, ^33^ which was comparable with our findings. These differences may be due to variance in the geography, sexual and personal behaviour of the patients.

The trend of ectopic pregnancy shows in 2016 a peak in February and a sharp drop in March, it peaks again between July and August. In 2017 the peak months were between January and February, a drop was seen with another sharp rise occurring between August and December. In 2018 the peak is seen in February and April.

The trends found in this study over the time period of two years (January 2016 to April 2018)shows that most cases of ectopic pregnancy occurred during the months of August to February, and the least cases were seen between March and July. The peak months for each year were not widely varied as August is the peak month for 2016 and October was the peak month for 2017 with February and April being the peak month for 2018. August to February accounts for most of the wet and cold seasons in the Gambia, so it could be expected that more sexual activity would take place during this period, leading to pregnancy and thereby giving rise to some cases of ectopic pregnancy. The periods with the low cases were attributed to dry seasons of the Gambia.

## Conclusion

Socio-demographic factors most affected by ectopic pregnancy includes age between 26–35 years and low parity (<4). A woman with low parity is more likely to have EP than a woman with high parity. The risk factors associated with ectopic pregnancy were pelvic inflammatory disease and previous history of abortion. Age and parity showed significant association with EP. Occupation had no association with ruptured and unruptured EP. It appears that the periods of high incidence of EP were seasonal more in the cold and rainy season.

## Recommendations

- Ectopic pregnancy is yet a significant contributor to the burden of maternal mortality. The most common risk factor being pelvic inflammatory disease which can be effectively treated if diagnosed early. The fact that it affects the majority of the young population makes it a public health issue.
- More focus should be put on sensitizing the community about ectopic pregnancy, the risk factors, the danger signs and the symptoms it comes with.
- Sensitize women and young girls on ways of preventing sexually transmitted diseases.
- Health workers should always screen for pelvic infections, sepsis and treat appropriately if discovered.

## Limitations of the study

The fact that patient's files from records were used means that there were issues with correct documentation, missing information and misplaced files.

## Figures and Tables

**Fig 3:**
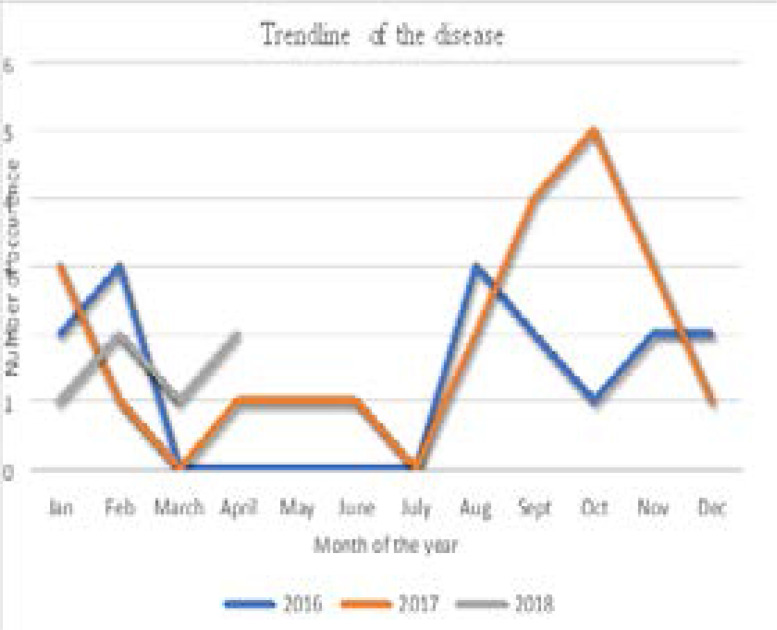
Trend of disease from year 2016 – 2018

**Table 1:** shows socio-demographic characteristics of the study population

	Frequency	Percentage (%)
**Age**		
**15 – 20**	4	9.0
**21 – 25**	9	20.9
**26 – 30**	12	27.9
**31 – 35**	12	27.9
**36 – 40**	6	14.0
**Marital status**		
**Single**	4	9.3
**Married**	35	81.4
**Divorced**	2	4.7
**Widowed**	2	4.7
**Ethnicity**		
**Wollof**	8	18.6
**Mandika**	11	25.6
**Fula**	4	9.3
**Jola**	12	27.9
**Others**	8	18.6
**Occupation**		
**House Wives**	37	86.0
**Government Workers**	6	14.0
**Residential Location**		
**Urban**	28	65.1
**Rural**	15	34.9
**Parity Level**		
**0**	6	14.0
**1**	14	32.6
**2**	8	18.6
**3**	2	4.7
**4**	6	14.0
**5 and Above**	7	16.3
Total	43	100

**Table 2:** shows risk factors

Risk Factors	Frequency	Percentage (%)
**Pelvic inflammatory disease**	12	27.9
**Previous ectopic pregnancy**	0	0.0
**Intrauterine contraceptives**	2	4.7
**Previous induced abortion**	10	23.3
**Previous pelvic surgery**	2	4.7
**Not stated**	17	39.5
**Total**	43	100.0

**Table 3:** shows the relationship of occupation with REP and UEP

	Housewives	Government workers	p-value
**Unruptured**	7	2	0.421
**Ruptured**	30	4	
**Total**	37	6	

**Table 4:** shows the relationship of parity and age to frequency of EP

Age	Ectopic pregnancy	p-value	
**21–25**	9	0.000	23.203
**26–30**	12		
**>35**	6		
**Total**	27		
**Parity**			
**<4**	30	0.001	
**>4**	13		
**Total**	43		

## Data Availability

The datasets generated and/or analysed during this study are available from the corresponding author on reasonable request.

## Abbreviations

EFSTH: Edward Francis Small Teaching Hospital
EP: Ectopic Pregnancy
PID: Pelvic inflammatory disease
REP: Ruptured ectopic pregnancy
SDG: sustainable development goals
UREP: Unruptured ectopic pregnancy
WHO: World Health Organization.
